# Looking back on childhood experiences of homelessness: Stories of ongoing residential instability and resilience

**DOI:** 10.1002/ajcp.70096

**Published:** 2026-08-10

**Authors:** Marybeth Shinn, Emma J. Sacks, Kayla Fike

**Affiliations:** ^1^ Department of Human and Organizational Development, Peabody College Vanderbilt University Nashville Tennessee USA

**Keywords:** childhood homelessness, doubling up, hardship and resilience, homeless shelters, racism in homeless services, social relations

## Abstract

Families' experience of homelessness is typically examined from the perspective of parents during or shortly after a shelter stay. Parents complain about rules, surveillance, crowding, and challenges to parenting in both homeless shelters and in doubling up with other households (sharing the others' homes), and relief when they attain their own place. The current study uses qualitative interviews to examine the retrospective views of housing and homelessness among young adults who were homeless as children with their families. We compare their perspectives to earlier reports of parents' contemporaneous experiences of similar settings. The 22 young adults interviewed in 2024–25 were children in families recruited from 16 homeless shelters in 10 states in 2010–2012 by the Family Options Study. They share many of parents' perspectives but focus on largely positive social relationships in all housing situations. They report ongoing housing instability, difficult experiences of racism in housing programs, and landlords who forced moves, but also their parents' and their own coping and resilience in the face of hardship. Policy implications include the need to address racial equity in the homeless service system. Reducing the shortage of affordable housing would not only reduce homelessness but also give households more leverage with landlords.

## Highlights

- Young adults' retrospective description of childhood homelessness shows hardship and resilience.
- Children and parents have similar views of homelessness but children focus on social relationships.
- Housing instability persists after families' stays in homeless shelters and extends into adulthood.
- Landlords and racism in the homeless service system contribute to housing difficulties.

## INTRODUCTION

Just over a third (33.6%) of people counted by the Department of Housing and Urban Development as homeless on a single night in January 2024 were members of homeless families with children (*n* = 259,473), the largest number ever recorded (de Sousa & Henry, 2024). Thankfully, most of them were sheltered. Approximately three and a half times as many families stay in homeless shelters over the course of a year (Henry et al., 2024). A much larger group of families, including 1,548,191 public school children, experienced homelessness in the 2023–2024 school year by the Department of Education's broader definition, which includes doubling up or living in another household's home (National Center for Homeless Education, 2025).

What is the experience of homelessness like for families, and what happens to them afterwards? A study using shelter records in four jurisdictions found that most families who leave homeless shelters do not return over the next 2 to 3 years (Culhane et al., 2007), but the findings are by now dated. More recent work (reviewed below) suggests that families continue to experience considerable residential instability (not primarily shelter use) over comparable or even shorter periods. Studies that interview parents (also reviewed below) describe the experience of staying in shelter and subsequent instability, again over a few months or years. There is little research that follows families for longer periods or gives voice to children. Understanding experiences over the longer term and including children's voices can help programs and policymakers to design better interventions.

The current study examines how young adults in their early twenties recall childhood experiences of homelessness and housing instability more than a dozen years after they entered shelters with their families. It describes shelter stays, subsequent interventions by the homeless service system, and families' continuing struggles to maintain housing over the next decade and more, all through the eyes of adult children. We ask to what extent children's memories of their experiences echo and diverge from those of parents, whether housing instability persisted over time, and whether respondents continued to experience homelessness and housing instability in adulthood.

## LITERATURE REVIEW

### Parents' experiences in homeless shelters

Previous studies of the experience of homelessness among families typically involve interviews with parents during or shortly after a shelter stay. A systematic review found that parents often felt stressed and inadequate because they could not meet children's material needs. Shelter rules and constant surveillance challenged their autonomy and self‐efficacy, staff usurped parenting roles, and parents felt unsupported. Nonetheless, the research described parents' adaptive strategies, strength and resilience, including efforts to shield children from the emotional difficulties resulting from homelessness (Bradley et al., 2018). The largest study in this group (Mayberry et al., 2014) interviewed 80 parents who participated in the Family Options Study, which is also the source of the sample for the present research.

Later studies echo these findings. Parents felt that physical conditions in shelters, including crowding and lack of privacy, contributed to their own stress and had adverse effects on children's mental health (Marçal et al., 2020; Sylvestre et al., 2018). Parents observed children's internalizing (sadness, anxiety) and externalizing behaviors (acting out, disobedience), along with sleep disturbances, and developmental regression (Choi & Snyder, 1999; Anthony et al., 2018; Owens et al., 2022; Sylvestre et al., 2018). Parents described restrictive rules, loss of autonomy, disruptions to family routines and parenting, and experiences of prejudice and discrimination based on race, sexual orientation, and gender identities. They also described ways to resist, strategies to manage the challenges, efforts to protect children from bad influences, and attempts to help them gain resilience (Fraenkel, 2020; Mayberry, 2016; Owens et al., 2022; Reppond & Bullock, 2020; Sylvestre et al., 2018; Williams‐Arya et al., 2021).

Although many parents felt disempowered (e.g., Anthony et al., 2018), this was sometimes balanced by gratitude (Choi & Snyder, 1999). In a quantitative analysis, perceptions of shelter community and lower levels of parental stress were positively related to caregivers' ratings of children's socio‐emotional functioning (Vrabic et al., 2022).

One study interviewed shelter staff to assess the developmental appropriateness of shelter spaces, programming, and staff knowledge and training (Halverson et al. 2022). Most of the 64 geographically diverse shelters had a dedicated space for children although staff often reported barriers to access. A majority of shelters offered programming related to parenting or parent–child relationships. Staff also described their own needs for training.

### Adolescent views from a homeless shelter

We identified only one study interviewing adolescents about living in a homeless shelter with their families (Reeves, 2021). The sample included 10 adolescents aged 14–17 from 7 families living in a single shelter and 3 others in temporary housing. Using photo elicitation, the author explored the teens' experience of homelessness and coping strategies. Adolescents emphasized the importance of family, friends, and sometimes additional trusted adults. They also identified internal resources, such as hope and optimism, that helped them to cope with being homeless. The shelter where they lived provided for basic needs “which allowed them to develop a sense of belonging and love within their family and shelter community” (p. 213). This study did not examine housing trajectories over time.

### Housing stability after shelter

A systematic quantitative review of women's post‐shelter experiences, which included 13 studies with at least some mothers in families, found that families continued to experience housing instability after leaving shelters (Jacobsen et al., 2024). In two observational studies, 28%–45% of families returned to shelter within 1 to 3 years after exit. An intervention study found that only 77% attained stable housing within 3 years, and four small pre‐post studies found that 66%–80% of families maintained their initial housing for 6 to 9 months following shelter exit, suggesting considerable mobility (Jacobsen et al., 2024). Gubits et al. (2018) found that over 2000 families in the Family Options Study became more stable between the 20‐month and 37‐month follow‐ups after recruitment in homeless shelters. Nevertheless, among those who received no special housing intervention, 35% had still been recently homeless or doubled up (living in someone else's place) as of the longer‐term follow‐up. This number was halved among families who received priority access to long‐term rental subsidies. This study did not examine instability over the entire 37‐month period, and no study in the Jacobsen et al. (2024) review had a longer follow‐up period.

### Adults and parents' observations about doubling up

There are fewer studies of the experience of doubling up. A study of adults 50 and over (with no mention of children) who punctuated spells of homelessness with stays with family or friends found many relationships of mutual support but also conflict between participants and their hosts. Participants felt shame about burdening hosts and felt stigmatized and judged by them. Sources of conflict included rules and boundaries and concerns on both sides about drug use (Knight et al., 2022). Families who doubled up prior to entering shelter experienced stress and tension and felt their hosts did not have the resources to support them (Choi & Snyder, 1999). Families in the Family Options Study who doubled up with others after shelter stays found both support and discord (Bush & Shinn, 2017). Although sharing homes is a common practice among poor households (Richard et al., 2024), doubling up after an episode of homelessness seemed predominantly negative. Parents reported interpersonal tensions, undesirable or even dangerous environments, and harmful effects on children. Parents felt uneasy even in welcoming and comfortable homes because the homes were not their own (Bush & Shinn, 2017).

### Parents' reflections on their own place

In a companion piece to Jacobsen and colleagues' (2024) quantitative review, the same authors reviewed qualitative experiences of women leaving shelter (Jacobsen et al., 2025). Finding their own place often involved families in multiple stressful moves, sometimes into housing that was unstable or dangerous. After successfully securing housing, women were “able to gain momentum, focus on future goals and …family stability” (p. 5). Feelings of independence, agency, autonomy, and control increased. Nonetheless, stigma and discrimination, and in some cases shame, sometimes followed women from shelter into housing. Most studies in this review interviewed women within 2 years of shelter exit.

Moving into their own place, typically with the help of a subsidy, brought parents in the Family Options Study an almost palpable sense of relief (Fisher et al., 2014). Increased independence and autonomy reduced parental stress and allowed them to re‐establish family routines. This, along with additional space, privacy, stability, and expectations of future stability in homes and schools had positive effects on children (Brown et al., 2023; Mayberry et al., 2014). Interviews in this study occurred 6‐8 months after families entered shelters (indeed some families were still there), so although parents sometimes worried about future instability or hoped to move after settling for a less‐than‐ideal place, their post‐shelter housing histories were short.

### Research questions

Previous studies have examined experiences of homelessness and instability over a few months or a few years, and rarely give voice to children. This current study describes young adults' memories of the homeless shelters where they stayed with their families as children and their continuing housing experiences through adolescence and early adulthood. We examine three questions:

1. To what extent do young adults' retrospective views of different housing situations match or diverge from parents' experiences in prior studies? For example, in shelters and transitional housing, were rules and restrictions as important to children as they were to parents? In doubled‐up situations, where parents chafed at interference with parenting, might children have formed lasting relationships with other adults? Were children aware of parents' efforts to shield them from the negative aspects of housing insecurity and homelessness? Did children, like parents, experience great relief when they obtained independent housing?
2. To what extent did families continue to experience housing instability leaving shelter?
3. Did children who had been homeless with their families continue to experience housing instability and homelessness in early adulthood?

## METHODS

### Participants

Twenty‐two young adults who had stayed in homeless shelters with their families 13–14 years earlier participated in semi‐structured descriptive interviews that asked about experiences of homelessness and subsequent housing up until the present time. Respondents included 10 males, 11 females, and 1 who identified as non‐binary. Ten identified as Black, five White, one each Hispanic and Asian/Pacific Islander, three mixed race, and two other. Ages at the time of the interview ranged from 20 to 30, with a median of 22. Education levels ranged from less than high school to midway through a STEM Ph.D. program. Respondents' families were recruited from 16 homeless shelters in 10 states, and the young adults lived in 15 different states at the time of the interview.

Respondents' families entered homeless shelters from 2010 to 2012 and were randomized to priority access to one of three housing interventions (long‐term rental subsidies, short‐term subsides, service‐rich transitional housing) or to usual care as part of the Family Options Study (Gubits et al., 2018). Usual care typically involved longer stays in shelter followed by whatever families could obtain, often later access to one of the other programs (Gubits et al., 2018). Not all families who were offered priority access to an intervention took up that intervention, and families in all conditions used a variety of housing programs over the years. For example, of families assigned to usual care, 21% had used a short‐term rental subsidy, 30% had used transitional housing, and 37% had used a long‐term rental subsidy at some time in the 3 years after entering shelter (Gubits et al., 2018).

The current study sampled young adults in families who used their assigned intervention at study outset, including nine assigned to long‐term rental subsidies, three to short‐term rental subsidies, six to transitional housing, and four to usual care. The larger study found substantial benefits of long‐term rental subsidies for housing stability and family well‐being 3 years after randomization but few differences among the last three groups (Gubits et al., 2018). For this reason, we oversampled young adults whose families received immediate access to a long‐term rental subsidy.

At the time of a follow‐up interview 12 years after study enrollment, parents were asked to provide contact information for adult children who had been with them in shelters at the outset. Participants in this study were referred by parents, completed consent forms and a web survey, agreed to future contact, and were recruited to the qualitative study, where they again provided informed consent. IRB approval was obtained for each stage of sampling. Because of fall‐off at each stage, participants in the current study are not representative of children who were part of the initial study. Contact information given by respondents to the web survey was frequently unstable 1 to 2 years later, with 27% of letters sent by US mail undeliverable, and 28% of phone numbers no longer valid. Information about others who would be in contact with potential respondents was similarly unreliable.

### Interviews

Using a semi‐structured interview protocol, respondents were interviewed individually over Zoom during 2024 and 2025 by trained graduate or senior undergraduate students—approximate age peers of respondents—including some with prior experience of homelessness. Interviews lasted a mean of 41 min (range 21–71). Designed by the first and last authors, the interview protocol covered respondents' memories, if any, of the initial shelter where families entered the Family Options study, any additional experiences of homelessness, transitional housing, doubling up with other households, and attaining, and often losing, housing of the families' own, up until the time of the interview. We asked about good things and bad things in each setting, instances of separation from parents, and any times when respondents had experienced homelessness or doubling up on their own, without parents, before or after age 18. We also examined young adults' perspectives on whether race or other social identities affected their experiences, and their attributions for housing instability. Other sections of the interview, addressed in other papers, examined respondents' views of successful adulthood and of good neighborhoods. The protocol is included in an online appendix.

Recognizing that childhood memories might not be reliable, we gave respondents permission to say they didn't know. Our first interview question was, “When we first met your family a dozen years ago, you were staying in [shelter name] in [city, state]. That's a long time ago. Do you have any memories of staying there with your family?”

Recordings of interviews were transcribed using automatic transcription software and corrected and de‐identified by interviewers who also annotated respondents' expressions or tone of voice when this seemed relevant to interpretation. After analysis, the audio recordings were deleted, and per agreement with the Department of Housing and Urban Development, paper contact information for participants was shredded, and additional information about respondents not supplied in the interviews was deleted. Deidentified transcripts are stored on a password‐protected and encrypted cloud service.

### Analysis

Drawing from qualitative data analysis techniques described by Tesch (1990, pp. 86, 91), and an essentialist approach to thematic analysis (Braun & Clarke, 2006), we sought to understand participants' recollections of their experiences, recognizing that their reports of childhood experiences are shaped by meaning‐making in the intervening years. The first two authors (a faculty member and a trained graduate research assistant who conducted many interviews) did independent open coding of the first section of a subset of interviews to identify themes, and the housing situation in which those themes were mentioned. The two analysts met to develop a consensual coding scheme in Excel. Both analysts then coded each interview, meeting every few days to modify the coding scheme as necessary and resolve disagreements by consensus. Next, the first analyst conducted constant comparison analysis, in which each instance of a theme is compared to all other instances, both to assure that codes were applied consistently and to examine variation in the experiences reflected in each theme (Lincoln & Guba, 1985; Strauss & Corbin, 1990). Most themes reflect an essentialist tradition of thematic analysis, aligning closely with the semantic level of participants' accounts and meaning‐making. Additionally, we developed theoretical, semantic themes that attended to the relational and emotional aspects of experiences and participants' descriptions of their emotional trials and triumphs, during both childhood homelessness and subsequent housing instability. The first analyst kept memos focused on parallels between the themes identified by the young adults and themes identified by parents in earlier studies, as well as more themes that answered the research questions. The second analyst reviewed responses to a later interview question that asked respondents about ongoing relationships with people with whom they doubled up as children. In service of the deductive aspects of the themes of doubling up and social relationships, this step examined whether respondents who had stayed with others in childhood maintained supportive relationships with those people. Relationships were considered supportive only when respondents specifically described them as such. Credibility of the findings was enhanced by continual monitoring of interrater reliability and peer debriefing amongst the analysts.

## RESULTS

To answer the first research question about how young adults' retrospective views of different housing situations match or diverge from parents' contemporaneous descriptions, we describe young adults' memories of shelters and transitional housing, doubling up, and moving into a place of the family's own and cross‐cutting themes. To answer the second question about ongoing stability, we look at the number of places young adults explicitly remember, some of the reasons for instability, and feelings of precarity. Then we turn to experiences of homelessness and doubling up in adulthood to answer the third research question on that topic.

### Shelters and transitional housing

Only one respondent, a teenager when she was homeless, differentiated between shelters and transitional housing. Although study records showed that six families stayed in the transitional housing programs to which they had been assigned by the study, and others stayed in these programs later, respondents typically did not recognize the term transitional housing and described shelters and transitional programs in the same ways. Thus, we consider them together.

Often respondents' families stayed in more than one shelter or transitional program over time. We did not collect full housing histories (and memories were sometimes hazy), but respondents described at least 38 shelter or transitional programs across all interviews.

In many respects, respondents' views of homeless shelters and transitional housing programs mirrored those expressed by parents. Many respondents were simply grateful for shelters:

*…having a place to stay in general, because obviously if we didn't, we would probably be on the street somewhere having a harder time, or maybe just sleeping in the car until we get back on our feet*. [respondent #7, male]

Shelter conditions varied from private rooms for a family to more challenging congregate spaces, with extreme cases involving bedbugs or sleeping three children to a bed or on mats on the floor.

*You're sharing a lot of stuff with other people you don't know. It was a lot of drama. I mean, obviously you are laying your head with people that are from the street, probably drug issues*. [#17, female]

*it wasn't a very comfortable feeling to shower at the homeless shelter, especially just by myself*. [#13, male]

Like parents, young adults recall problematic shelter rules. Some shelters did not permit men or older boys to stay with families. Rules that children had to be continually supervised by an adult made it difficult for a mother of an 11‐year‐old to go to work [#18, male] and restricted families' movements:

*You just have to constantly be inside the building at all times. You're not really allowed to leave, and then you have to get permission to play on the playground that's like right outside*. [#16, female]

Respondents resented rules requiring families to leave shelters during the day or curfews:

*So, if I wanted to stay over at my friend's house or like my cousin's house, I couldn't because we had to go back. Because if you didn't get in by a certain time, … you couldn't go inside*. [#9, female]

*You couldn't stay there all day. So, for us, we had to be out of there early in the morning. I guess so they could sanitize the facility. So, every day we had to just go somewhere, and it made it challenging, especially on weekends where we didn't have a place to really stay*. [#12, male]

In other respects, respondents were far more positive than parents about living in shelters. Young adults had fond memories of activities and services they and their families received:

*We had movie nights sometimes at our room and other people's room. I can't really think of anything bad. They had a computer, like a learning area. They had a TV area. They had a park*. [#21, female]

*I remember the [shelter] helping us a lot, especially during Christmas. I used to get very excited about the presents that they would provide us*. [#8, male]

*It was mostly for getting my mom on… food stamps and stuff like that … they would help us get glasses. So there was a lot of things that we didn't have that they helped us get*. [#2, male]

Some young adults who stayed in shelters with a religious mission found the experience transformative in ways that shaped later life.

*Well, me and my family got more in touch with our faith. There was a cookout one day and we got invited to a church, and ever since then, we've been going to this church. My entire family, household wise, has been baptized at this church. So, I thank God for bringing us in and letting us know that he is still by our side*. [#12, male]

### Doubling up

All but three of the respondents described experiences in which their family had doubled up with family or friends. Across 22 respondents, there were at least 38 instances of doubling up with their families, sometimes before but most often after the family joined the study. Many stays were brief, interleaved with other forms of housing. Some extended for years. Families most often doubled up with extended family members, but sometimes with friends.

Doubled‐up households, like shelters, were sometimes overcrowded and “a little hectic” [#14, female], offered “no personal space,” and required respondents to adapt to hosts' “personalities.” [#21, female]. Respondents mentioned sleeping on air mattresses, on a blanket on the floor, or sharing beds with other children. Some doubled‐up situations were potentially dangerous:

*[A] roommate that [was] super obsessed with, like, looking at us naked. It wasn't like he touched us or anything, but he was, like, always stalking outside the bathroom. And my mom hated it, so she knew we had to get out of there*. [#3, female]

*[The mother's friend] talked a lot about sexual stuff around kids, exposed us to porn. She put her hands on me several different times*. [#20, non‐binary]

But overall, positive interactions outweighed negatives in respondents' narratives. For example, one respondent downplayed fighting among adults in an experience deemed positive overall.

*[Staying with uncle] was good, I guess. But my mom and my dad and my aunt would get into fights a lot. Because my aunt really didn't like my mom at the time. So, it was. It was a lot. But you'll have drama and families. So not every family is perfect. [Interviewer: Was there anything… positive that stands out to you?] Just spending time with my family. And just being with the people that I, that loved me and the people that I loved*. [#15, female]

Respondents consistently described happy memories and strong familial or kinship relationships.

*No, I just have a few happy memories, like Christmas time, opening gifts*. [#4, male]

*But I think we just liked being with our grandma. And I think it was really cool being with my cousins and hanging out with them and yeah, it was a really nice experience, I guess*. [#9, female]

Relatives sometimes provided help or material resources beyond housing:

*When I was living with my aunt, it was a lot of help for my mom and my dad because they was able to go to work and not have to worry about a daycare, or who's going to take care of the kids. Because my aunt was at home*. [#21, female]

Some young adults had lost touch with people with whom they stayed, or relationships with grandparents were disrupted by death. But many reported positive relationships in adulthood with people with whom they had doubled up as children.

*I would have to say my grandparents is the biggest help… helping me how to do right at work, how to spend money, how to save, and how to… what do you call it? How to let your actions speak more than your words* [#18, male].

Other relationships were positive, but less effusively so:

*She lives pretty far away now, but we still are in good contact with each other. I don't have no real bad energy with none of my aunts. So, every time we see each other, it's highs, hugs, kisses. And then …we catch each other up and stuff like that. So, everything's the same. It's like it's normal*. [#21, female]

### Own place

Families of all but 1 of the 22 respondents eventually moved into their own place. Young adults often knew whether their families were getting help with rent, but they rarely differentiated between short‐term “rapid rehousing” assistance and longer‐term vouchers. Like parents, adult children mentioned that some of the places they lived in were in poor condition, with problems with flooding, plumbing, bugs, mold, and a place “falling apart” [#16, female]. And like parents, they often expressed relief at getting housing, which meant additional space, freedom, and stability.

*It was such a relief to finally have my own space and not be so surrounded with people who I couldn't necessarily trust. It was very. It was such a relief….I had, like access to my old toys, which brought a lot of comfort. I was definitely more of a comfort thing. I felt a lot safer to shower at home*. [#13, male]

*We could cook and eat when we wanted to. We didn't have to eat at a certain time. Schooling was much easier where it wasn't a stress on my mind*. [#12, male]

### Cross‐cutting themes

Social relationships were far more salient in young adults' memories than in parents' accounts. Descriptions of family resilience in the face of hardship correspond more closely.

#### Social relationships

Relationships with peers were important across housing types. In shelters:

*Like when you're a kid, you just kind of focus on, like, the kid part of it, meeting other kids*. [#13, female]

In doubled‐up situations:

*I had this best friend over there… We kind of grew up together, and you know, it was fun. There was a lot of people, a lot of people, a lot of kids my age, you know?* [#14, female]

And in their own place:

*So, we all grew up doing some fun, cool stuff, riding our bikes down the hill, or playing games at each other's houses*. [#18, male]

Not all relationships were positive. Respondents sometimes experienced stigma at school, most often while staying in a shelter.

*The people at the school were particularly like, not particularly nice because they knew where we were coming from*. [#13, male]

*There were several encounters where I can recall that my clothes may have been wrinkled and stuff and a couple of kids, you know, picking on me or whatever for that*. [#12, male]

Relationships with adult neighbors (in own housing and doubled‐up situations) were mixed.

*People would give you, like, a certain look, like they're actually judging you from afar. And then some*. [#18, male]

*But our neighbors like to the right of us was an elderly lady that we would always hang out with, always talk with. And even everybody across the street was, they were all so nice*. [#4, male]

#### Family coping and resilience amidst hardship

Young adults were acutely aware of their families' financial struggles, but they also gave credit to their parents' efforts to create a better life for their families. This included efforts to safeguard children from problems in shelters:

*A lot of nights my mom didn't sleep … she would kind of just stay up and watch us and watch our things*. [#17, female]

*There was a few people there that wasn't super comfortable around… So, it was a little weird, but… my dad did pretty well at keeping us away from them*. [#19, female]

Parents tried to protect children from unsheltered homelessness.

*When we stayed with [my Dad], he would get a hotel so that we're not sleeping in the car… he was sleeping in the car when we weren't there*. [#19, female]

In addition to these efforts to shield children from the worst aspects of homelessness, parents sought to extricate their families from homelessness and poverty altogether:

*We were still struggling to get by to really put food on the plate, that's for sure…. My Mom… did end up having to switch to new jobs just to make ends meet*. [#10, male]

*And my mom was trying to get back on her feet because she came here with a couple of suitcases and two children. So, she didn't have much. So, she was trying to work. She was going to school*. [#12, male]

Parents also tried to shield children emotionally, not always with complete success:

*So, we were doing what we can. I know my parents did their best to not show their stress. They did the best they can, but obviously as a kid you still feel that in some way. So, I know my parents are working hard to figure out the next situation*. [#11, male]

Parents in the Family Options Study spoke of protecting children from homelessness and hardship by sending them to live with other relatives (Shinn et al., 2015). Four respondents in the current study described a total of six instances when they lived with another household, most often grandparents, without their parents. In one case, a family negotiated to have different children live with three different relatives [#13, male]. Adult children described the facts of these arrangements, but they rarely spoke of parents' motivations. An exception was:

*Things definitely changed as soon as my mom told me to go with my grandparents [Interviewer: how did that change come about?] Uh, I think my mom was like, it'll be better, for you to just live with the grandparents so that you can be raised more differently than the way I would want you to be raised*. [#18, male]

Grandparents could also be a source of stability when parents were unable to provide that:

*Probably from the age of like when I was born to five, I stayed with my mom and my grandpa and everybody, but she [Mother] wasn't presently in my life. She'd run off a lot. So, I wasn't, I didn't really have her in my life that much*. [#14, female]

### Ongoing instability

Our second research question asked about the extent to which housing instability continued in the years after an episode of homelessness. Counting only places explicitly mentioned by respondents, the young adults described staying in at least 6.4 places on average or a move every two or so years. Three families spent time in hotels in between other living situations, one stayed in a car and on the floor of a store. Some respondents could not recall the number of places they stayed.

*We were getting help from the homeless shelter, and we were basically surviving…. I mean, we bounced from houses to houses* [#8, male].

*We would go from one school for like a few months and another school and another school*. [#4, male]

Instability led to feelings of precarity:

*With those experiences, it feels very like you have to take advantage of everything while you have it, because you never know when you're going to lose it. And once you lose it, it's going to take more time to work up to gaining all of those things back again…And just like the fear of like being out on the street again is, it's like constant with my family*. [#16, female]

*But I guess the only con that would come with that when you're coming out of not being stable, is always feeling like you're going to be unstable again*. [#1, female]

Even places of the families' own were often insecure. The 22 respondents lived in at least 50 houses and apartments of the families' own, that is, not doubled up with others. Often, families could not afford the rent:

*I think it was the building owner just kept raising the rent and we could no longer afford it. And so we went*. [#13, male]

In four instances, landlords also forced families out, often quite abruptly:

*The owner of the house had to sell it, if I'm right. So, he had to sell it. So, we were pretty much forced to move out anyways. And we were looking for a house for a while too, and we couldn't really find one actually*. [#10, male]

*They just wanted to get rid of the house where we stayed at. They wanted to get rid of it so we couldn't lease it again…. They wanted to destroy it…They were like oh, because they were adding, they were adding new houses and they're bringing in new housing*. [#6, female]

*[After a son inherited a property from a previously helpful landlord,] at some point, he said,” okay, I sold the house. You have 2 weeks to find a place. Get out.”* [#4, male]

*And then the leassor moved his sister into our house and evicted us…He had. Yeah, it was really an insane time. We basically had I think it was 24 hours to gather all of our things and leave that place*. [#13, male]

Sometimes there were disputes between the families and their landlords, although young adults were not always clear on the reasons:

*Something happened and we had to do a lawsuit or something*. [#19, female]

In one case, a landlord kept the family's furniture, claiming falsely that the apartment had been furnished when they moved in. [#20, non‐binary].

**Housing search and social identities.** The instability meant that many families had to search repeatedly for housing. Like parents in the Family Options Study (Fisher et al., 2014], the young adults said that families sought affordable places where the whole family could be together, close to parents' jobs and family support systems. We asked whether parents' social identities affected the process of obtaining and maintaining housing. Nine of 22 respondents, six Black, one Hispanic, one mixed race, and one White, said yes. Black respondents felt that Whites were moved from waitlists faster and were offered housing in better neighborhoods:

*And they had gotten there after us and then also got out of there way before, like way before us*. [#14, female]

*And most of the times it was just like, if you're being rehomed, you're going to be in a majority Black neighborhood, that that's it. Yeah, I've noticed with my mom she had a friend that she made when we lived in the shelter and it was a White woman, and it was like they got rehomed into a nicer neighborhood. I would say more in, like, the suburb area. And then it was just with me and my family, we were just put in the city*. [#16, female]

The mixed‐race woman described the same phenomenon from the other side:

*I feel like it was easier because we're White. Us kids, except for my older brother, were [mixed]. So that kind of made it easier… But if I feel like if you're White, you get pushed up on the list, and that could be wrong. But that's how I feel about it*. [#3, female]

Two respondents saw financial limitations as more important than race or ethnicity:

*I would say a little bit, but not mainly. I feel like it's mainly just based on money. If you have the money, you can get it*. [#7, male]

### Doubling up and homelessness in adulthood

Our third research question asked about ongoing housing instability in adulthood. A majority of our respondents (14 of 22) experienced homelessness or doubling up on their own after age 18. Seven respondents reported that they had been homeless as adults, without their families. Two of them slept in cars and one in “a dark, unfinished construction area [#18, male].” Six respondents (including the two who slept in cars) identified as homeless while living doubled up with friends, extended family, or even parents for periods ranging from a few weeks to years:

*I had to sleep in the living room. It was awkward because my parents sleep in the front room, they like sleeping there. So, we basically just shared a giant room together*. [#3, female]

Three additional respondents who did not identify as homeless described doubling up as adults. Those descriptions were somewhat more negative than descriptions of doubling up as children. Respondents mentioned a lack of space and privacy, and difficulty navigating relationships and shared spaces with other people. At the same time, respondents expressed gratitude for being taken in.

*I'll say like, being told what to do. Like cleaning up behind grown people… and it's not your mess*. [#6, female]

*Not knowing whether my stuff was going to get stolen when I'm asleep, whether these people are going to trip on us and not let us back in the house when we need to come back, just a whole bunch of stuff*. [#14, female]

*His parents were nice enough to drive from Tennessee all the way to at the time I was trying to live in Texas. And they let me stay with them until my boyfriend got out of the military…It was nice. They were, they were nice*. [#15, female]

Five respondents, all Black women, said their identities affected their housing options as young adults:

*But as far as and me being Black as well just does not help. So, you know, yeah…Like I said, well, my felony and credit and just me being Black on top of that, it just doesn't help. It's probably. I feel like I don't know. I don't know*. [#14, female]

*Yeah, definitely me being a Black, young woman. I can definitely say I'm overlooked for a lot of things. Like when I walk into a nice building. A leasing office, automatically, they're like, “Well, you have to make this much.” And it's like, “Okay, who said I didn't make that much?” It's a stigma there*. [#17, female]

**Personal resilience.** Respondents also described their own personal growth and resilience:

*I'm very happy I finished school, I got my GED and my high school diploma. And so I'm just slowly trying to build my life, and trying to right now, it's like I'm just trying to go, see what's out there, trying to see what life has. But from my childhood, it's been a rough roller coaster, but I think I've made it. I think I've done well for myself so far*. [#2, male]

*I guess I could say one thing positive came out of [instability. It] kind of grew me strong bones where I can adapt to situations, but I also feel like that's a negative because I can adapt in the worst situations because I've had to do that in my life, even though you shouldn't have to get used to something so horrible*. [#17, female]

In sum, young adults painted a picture of continuing housing instability, first with their families and then on their own during over a dozen years after childhood stays in homeless shelters with their families. They describe continuing hardship, but also familial and personal resilience, and focus on the importance of social relationships. Respondents also noted ways that landlords and housing programs contributed to housing instability.

## DISCUSSION

In many ways, young adults' memories of their experiences of homelessness mirrored earlier reports by parents in families experiencing homelessness. Like parents, respondents complained about physical conditions and rules in shelters and reveled in the space, privacy, and autonomy afforded by living in their own place. However, many had fond memories of shelters and the activities there. Perhaps because children are generally under adult supervision and lack autonomy, they were less aware of parents' experience of surveillance and usurpation of parental authority in shelters and doubled‐up situations. Although the young adults expressed gratitude when non‐family members took them in, they did not describe feeling a burden or feeling that the places were not their own. The young adults' descriptions of doubling up in adulthood are more like those of parents. They too expressed relief in moving into their own place but did not echo parents' reports that their own behavior improved.

Young adults described hardship and parental stress and admired their parents' efforts to shield them and create a better life for the family. Parents' accounts were more specific (e.g., calculating whether they could afford demerits for skipping a required meeting to take the child to a church youth group; taking a younger child out to give an older child private time in their shared room) (Mayberry et al., 2014). It is not clear whether respondents were unaware at that level of detail, or whether the specifics did not survive the intervening years in their memories. Young adults also talked about their own resilience and the ways that adverse circumstances led them to become more adaptable.

A key difference from earlier reports by parents but a similarity to the interviews with adolescents by Reeves (2021) was the centrality of relationships to young adults' recollections and evaluations of the places they lived. The young adults talked about positive relationships with other children, with family members and extended family, and with neighbors, whereas parents were more likely to talk about keeping to themselves as a coping strategy (Mayberry et al., 2014). Relationships were not uniformly positive—young adults recall being stigmatized, especially at school, and disagreements with landlords, but they also recalled having fun with other children, and feeling loved. In some cases, relationships from periods of doubling up persisted into adulthood, although it is not clear if this is more frequent than would have occurred without sharing a residence. Respondents gave credit to service providers and their parents for creating opportunities for relationships to blossom.

Young adults had a much longer view of families' ongoing instability than in any previous study. Although at 3 years, in the much larger survey sample, Family Options families who were assigned to priority offers of housing subsidies were more stable than families in the other three groups, group assignment did not matter over the long term in our small qualitative sample. Frequently, young adults could not remember all the places their families had lived; the estimate that families moved, on average, every 2 years is conservative, as we counted only places that respondents specifically described.

Respondents attributed some residential churn to landlords. Young adults may not have been privy to the objective details of parents' rent disputes with landlords, but recollections of abrupt evictions are likely accurate. In this way our results are more similar to, but more extreme than, studies of poor families who may or may not have ever lived in a shelter (Desmond, 2016; Goldstone, 2025; Orr et al., 2003) Two thirds of participants in the Moving to Opportunity [MTO] experiment had moved again within 4 to 7 years after using vouchers to move to low‐poverty area, with 22% citing leasing problems and 20% citing conflicts with landlords as a reason. As in this study, an unspecified number of landlords in the MTO sample “sold their buildings with little warning” (Orr et al., 2003).

## STRENGTHS AND LIMITATIONS

Data draw on the Family Options Study, the longest study ever conducted of families who experience homelessness. The facts that families were recruited from 16 shelters and that the young adults lived in 15 states at the time of the interviews suggest a broader picture of homelessness and its aftermath than in studies that sample from a single shelter or locale. But like most qualitative explorations, our sample is small and not representative of all children who experience homelessness with their families. Parents who were found and re‐interviewed 12 years after shelter entry may have been more stable than average. Similarly, respondents whose contact information had not lapsed 1 to 2 years after they completed a web survey were probably more stable than peers who could not be reached. Our respondents, even two who had spent time in foster care, may have had especially positive ongoing relationships with the parents who referred them to the study. Accounts reflect children's understanding of circumstances and events of which they were aware, filtered through the eyes of the adults they have become. This standpoint is both a weakness and a strength. Memories of long‐ago events can be faulty, but we focus on what was salient enough for children to recall years later. Young adults paint a picture of hardship and ongoing instability in the years after entering homeless shelters, but also of coping and resilience, and emphasize the central role of social relationships.

## RECOMMENDATIONS FOR FUTURE RESEARCH

Clearly, administrative data (primarily returns to homeless shelters) are insufficient to capture the extent of housing instability experienced by people (whether individuals or families) after shelter stays. Surveys or interviews can supplement these records. Quantitative surveys necessarily elide details of people's experiences, as do retrospective qualitative studies such as this one. Retrospective studies can be biased, as respondents recall information that makes sense of their current situation. And cross‐sectional studies, whether quantitative or qualitative, miss the dynamic processes that our study showcases. Pairing periodic surveys (ideally with large and representative samples) with periodic qualitative interviews to keep recall periods short and examining the perspectives of all members of households would address the limitations of previous research but would be both expensive and challenging (especially when the sample is defined by instability). More practically, retrospective studies that are anchored in documented experiences (here, the initial shelter stay) can provide important information. Targeted qualitative retrospective supplements to ongoing longitudinal surveys that document earlier experiences would provide a strong addition to the research literature.

## IMPLICATIONS FOR PROGRAMS AND POLICY

Respondents appreciated many shelters, but their accounts of the consequences of restrictive rules and lack of privacy may help service providers create better experiences for families. Respondents described discriminatory processes in housing programs that favored white families, suggesting a need to center racial equity in work to end homelessness. National studies show that Blacks and other minorities face ongoing (illegal) discrimination in the housing market (Turner et al., 2013), a fact that contributes to the overrepresentation of Blacks and Native Americans among people who become homeless (de Sousa & Henry, 2024). It is disheartening that the homeless service system sometimes compounds these forms of discrimination. A study by the Urban Institute documents landlords' discrimination against voucher holders (Cunningham et al., 2018), which can mask illegal racial discrimination. Communities need to enforce fair housing laws and pass new laws to prohibit discrimination against households that pay part of the rent with a voucher. Those laws make a difference, where they exist (Cunningham et al., 2018).

It is not news that the national shortage of housing for low‐income households has put rents out of reach for many poor families (Colón‐Bermúdez et al., 2025) and that rent levels are the primary correlate of city‐to‐city variation in rates of homelessness (Colburn & Aldern, 2022). It may be less obvious that the shortage of affordable housing puts poor families at a disadvantage in dealing with landlords, as demonstrated when landlords evicted families abruptly to turn their properties to other uses. Creating more housing affordable to families at the bottom of the income distribution is critical to end homelessness and increasing vacancy rates would offer poor households more leverage in the rental market.

Our study highlights children's ability to forge lasting friendships even in difficult circumstances. Creating opportunities for such friendships to flourish, for example, in recreation centers or community activities, could be a useful tool to integrate formerly homeless families and other poor families into mixed‐income communities where proximity alone does not always lead to mixing across class lines (Fraser et al., 2013).

## CONCLUSION

Residential instability among families experiencing homelessness lasts well beyond an initial period of shelter use. It often extends into adulthood for children who stayed in homeless shelters with their families over a decade earlier. Young adults who were previously homeless with their families describe their experiences in ways that parallel and diverge from previous reports from parents over much shorter periods. Both describe both hardship and resilience. The young adults are less sensitive to usurpation of parental authority and more attuned to social relationships. The ways that shelter rules sometimes compound hardship, the possible role of racism in the allocation of housing resources, and landlords' contribution to residential instability all suggest avenues for changes in programs and policies.

## CONFLICT OF INTEREST STATEMENT

The authors declare no conflicts of interest.

## ETHICS STATEMENT

The study was approved by the Vanderbilt University Institutional Review Board: RE: IRB #241343 “Adult Children of the Family Options Study.” All participants competed informed consent—statement available on request—electronically via REDCap.

## Supporting information

Supporting file 1.

## Data Availability

Because the data are qualitative interviews that are difficult to fully blind, they are not available
