# Supplementary material for: Looking back on childhood experiences of homelessness: Stories of ongoing residential instability and resilience
Source: Am J Community Psychol. 2026 Aug 10;78(1-2):295–308. doi: 10.1002/ajcp.70096 (PMC13614426; doi:10.1002/ajcp.70096)
Supplement: Supplementary file 1 [file AJCP-78-295-s001.docx]

Interview Schedule

*Part 1: Recollections of homelessness and study interventions. Because families in all groups often used multiple programs, we will ask everyone about all programs.*

**Thanks so much for agreeing to talk with me.**

**When we first met your family a dozen years ago, you were staying in [INSERT SHELTER NAME] in CITY, STATE. That’s a long time ago. Do you have any memories of staying at there with your family?**

Probes: What was that like? What was staying in shelter like? Were there good things? Were there bad things?

**Did you and your family ever stay in a transitional housing program?**

*[If asked:] A transitional housing program is a program that offers temporary housing and social services to families who have been homeless.*

Probes: What was that like? What was staying in a transitional housing program like? Were there good things? Were there bad things?

**Did you and your family ever double up, or stay with another household temporarily in their home when you didn’t have a place of your own?**

If yes, whom did you stay with? Probe: grandparents, other relatives, friends? How long did you stay there (was this a long-term arrangement)

Probe: What was that like? Were there good things? Were there bad things?

**Did you and your family ever move into a place of your own?**

Probes: What was that like? Were there good things? Were there bad things?

**People’s race and ethnicity often affects their experiences, including access to housing. Do you think that your parents’ race or ethnicity affected your family’s experience in obtaining and maintaining housing?**

Probe: How?

**Did you ever live apart from your parent(s) while you were a child?**
Probes: How did that come about? *[Note to interviewer: we are interested in housing issues and economic hardship. If the separation was unrelated, you can skip the probes.]* Where did you stay? What was that like? Were there good things? Were there bad things?

**Did you ever experience homelessness on your own, without your parents? By experiencing homelessness, we mean times where you didn’t have a regular place to live, and you were living in emergency shelter, transitional housing, or in an unsheltered location.**

Probes: When was that, were you already 18? How did that come about? Where did you stay? Were there good things? Were there bad things?

**Did you ever stay with a friend or relative or “couch surf” or “double up” with another household in their space on your own, apart from parents, because you didn’t have another place to stay?**

Probes: When was that, were you already 18? How did that come about? Where did you stay? Were there good things? Were there bad things?

**(If any of these) People’s social identities including race, gender, and sexual orientation often affect experiences like these. Do you think your social identities affected your living apart from family or housing experiences on your own?** Probe: How?

*Part 2: Successful adulthood and contributing factors*

**Now let’s talk about the present.**

Where are you living now? Probe: Do you live with other people? Who?

**People have a lot of different goals in life. Can you tell me for you what successful adulthood includes?**

Probes if not mentioned: Housing, education, employment, family, relationship with other people, independence?

**Do you feel you are on track to attain or reach your goals?**

Probes: Why or why not?

**What has gotten in the way of your attaining your goals?**

**What has helped you on your path to successful adulthood or helped you to overcome obstacles on the way?**

Probes: Where did those supports come from? Were there particular people who helped? [If so,] Do you still get support from xx? Can you still rely on help from xx if you need it?

**How do you think your housing situation over the years affected your path to achieving those goals?**

**People’s social identities including race, gender, and sexual orientation often affect their ability to attain their goals. Do you think they affected your path?** Probe: How?

*Part 3 Social and community relationships*

**Now I have some questions about your relationships with people inside and outside of your family.**

**What is your current relationship with your mother like?**

[*Interviewer: Use respondent’s definition of mother. If respondent asks,* Whom do you think of as your mother.] Probe: Have you stayed in contact over the years?

**How about your current relationship with your father. What is that like?**

[*Interviewer: Use respondent’s definition of father. If respondent asks,* Whom do you think of as your father.] Probe: Have you stayed in contact over the years?

**What is your current relationship with your extended family (e.g., grandparents, siblings, aunts/uncles, cousins)?**

Probe: ask specifically about any people with whom the respondent doubled up.

**Are there other older adults other than family members whom you can rely on for advice or help or just to lend an ear?**

**Are there people your own age whom you can rely on for advice or help or just to lend an ear?**

**Do you have children of your own?**

Probes: How many? What ages?

**Now I have some questions about neighborhoods…**

**How would you describe your current neighborhood?**

Probes: Would you say it is more urban or rural or something in between? What are some good things about your neighborhood? What are some bad things about your neighborhood? What is your overall opinion of the neighborhood?

**When you think about what makes a good neighborhood [for you and your family], what does that include?**

Probes: Which of the things you mentioned would you say is most important and least important?

*Examples:* Do you think about relationships with your neighbors? Do you think about activities to do in the neighborhood? What about where the neighborhood is in the city or town?

**How would you say your current neighborhood compares to your idea of a good neighborhood?**

**What aspects of your neighborhood make you think so?**

**How does your current neighborhood compare to the neighborhood where you spent the most time growing up?**

**These are all the questions I have. Is there anything else you want to tell me?**

**When we have finished all our interviews and have had time to think about what you have said, we will present what we have learned back to a group of interviewees who are interested to understand whether we got it right. Would you like to be part of that group?**

**Thanks so much for your time. I’ve enjoyed talking with you.**

**[Information about payment]**

**Interviewer: Note the length of the interview (in minutes) and add this to the transcript.**
